# Low Cost Interconnected Architecture for the Hardware Spiking Neural Networks

**DOI:** 10.3389/fnins.2018.00857

**Published:** 2018-11-21

**Authors:** Yuling Luo, Lei Wan, Junxiu Liu, Jim Harkin, Liam McDaid, Yi Cao, Xuemei Ding

**Affiliations:** ^1^Faculty of Electronic Engineering, Guangxi Normal University, Guilin, China; ^2^School of Computing, Engineering and Intelligent Systems, University of Ulster, Londonderry, United Kingdom; ^3^Management Science and Business Economics Group, Business School, University of Edinburgh, Edinburgh, United Kingdom; ^4^College of Mathematics and Informatics, Fujian Normal University, Fuzhou, China

**Keywords:** interconnected architecture, spiking neural networks, Networks-on-Chip, system scalability, arbitration scheme

## Abstract

A novel low cost interconnected architecture (LCIA) is proposed in this paper, which is an efficient solution for the neuron interconnections for the hardware spiking neural networks (SNNs). It is based on an all-to-all connection that takes each paired input and output nodes of multi-layer SNNs as the source and destination of connections. The aim is to maintain an efficient routing performance under low hardware overhead. A Networks-on-Chip (NoC) router is proposed as the fundamental component of the LCIA, where an effective scheduler is designed to address the traffic challenge due to irregular spikes. The router can find requests rapidly, make the arbitration decision promptly, and provide equal services to different network traffic requests. Experimental results show that the LCIA can manage the intercommunication of the multi-layer neural networks efficiently and have a low hardware overhead which can maintain the scalability of hardware SNNs.

## Introduction

The current understanding from neuroscience research is that the mammalian brain is composed of dense and complex interconnected neurons and exhibits many surprising properties, e.g., pattern recognition, decision making, and so on (Cios and Shields, 1997). One key outcome is a computational neural model of spiking neural network (SNN), which offers a closer approach to model biological neurons than previous artificial neural network (ANN) models (Gerstner and Kistler, 2002). SNN attempts to emulate information processing based on massively parallel arrays of neurons that communicate through the timing of the spikes (Carrillo et al., 2013). A spiking neuron consists of a cell body (soma), a neuron output (axon), dendrites, and synapses, and so on. When the post-synaptic membrane potential of a neuron exceeds a firing threshold value, it fires and generates an output spike to the connected synapses/neurons. This leads to a strong computing capability of SNN and the SNN is widely used to solve problems in various fields, e.g., forecasting (Park et al., 1999; Kulkarni and Simon, 2012), image processing (Perrinet, 2008; Charleston-Villalobos et al., 2011), retinal coding (Rast et al., 2008), multi-view pattern recognition (Wu et al., 2008; Wysoski et al., 2008), and so on. These applications generally require an SNN system containing a large number of neurons for the information processing and computation (Akopyan et al., 2015; Sabour et al., 2017). These neurons are interconnected in a complex pattern and communicate by the spike events (Carrillo et al., 2012), where the interconnected strategies, e.g., Networks-on-Chip (NoC), are usually used for the communications between neurons of the hardware SNNs. Research shows that the communication mechanism should be carefully considered during the hardware development (Schuman et al., 2017). These interconnected strategies should be efficient in hardware and also support various SNN traffic statuses, e.g., regular and irregular spike events (Gerstner and Kistler, 2002). In addition, the required hardware area of the interconnected fabrics generally increases proportionally with the number of neurons and synapses. Thus a low cost interconnected architecture (LCIA) is very crucial for an SNN hardware system in order to support the system scalability, and is also very beneficial for the SNN to implement the models at high abstract level such as hierarchical temporal memory (Billaudelle and Ahmad, 2016; Walter et al., 2017).

The NoC paradigm was introduced in the approaches of Dally and Towles (2001); Benini and De Micheli (2002), and Jovanovic et al. (2009) as a promising solution to address the on-chip communication problems. It uses the computer network concept to achieve a similar network structure in hardware (Harkin et al., 2009). In general, the NoC system is composed of a set of processing elements, routers and links, which are arranged in a specific topology depending on the applications (Carrillo et al., 2013). It has been used for the hardware SNNs where the processing elements represent the neurons of the SNN and are connected by the routers and channel links (Emery et al., 2009; Harkin et al., 2009; Carrillo et al., 2012; Liu et al., 2015; Schuman et al., 2017). For example, the TrueNorth (Merolla et al., 2014) and Liohi (Davies et al., 2018) architectures use the routing networks (similar to NoC) for transmitting spike events. These approaches used either the baseline or some variations of the well-known mesh topology. The mesh topology consists of an *n*-dimensional array of nodes connected by a regular structure, where each node connects to its direct neighbors through north, east, west and south directions (Mohammadi et al., 2015). A NoC generator is proposed in the approach of Kwon et al. (2017) to generate a tailored NoC for the traffic flows in the neural network accelerators. Research showed that for the mesh topology, when the NoC size increases, the required fabrics for the interconnection increases which leads to a considerable hardware area overhead and prohibits the system scalability. In addition, for the common used feed forward neural networks, one neuron in a previous layer connects to all the neurons in the next layer. If it fires, it generates a spike and transmits it to all the connected neurons in the next layer (Mohammadi et al., 2015). This is a typical multicast transmission, thus for the hardware SNNs, to support the multicast communication is critical. With these motivations, this paper explores a novel LCIA for the SNN hardware systems. The LCIA is an on-chip interconnection fabric to provide an all-to-all connection method between different layers of SNNs, which gives a low hardware overhead and can maintain the SNN system scalability.

Preliminary results have been published in Luo et al. (2018) and each input port is associated with a traffic status weight, which is calculated based on the channel traffic and previous grant information. The router scheduler includes the weight calculation and comparison process, and it occupies 4.99% of the router area. In this approach, we go further and optimize the router design, especially the scheduler module. This approach is novel as the LCIA is an all-to-all interconnection strategy, which is well applicable for the multi-layer SNNs than the well-known regular topologies (e.g., mesh) (Carrillo et al., 2012; Liu et al., 2015, 2016). The LCIA employs a novel NoC router as the basic component where an effective scheduler is designed to address the traffic challenge under various spike patterns (i.e., regular, bursting, fast and rebound spikes, etc.) (Carrillo et al., 2012). The area utilizations and power consumption of this architecture are obtained using the Synopsys Design Compiler tool for SAED 90 nm CMOS technology. The results show that the total hardware area and power consumption of a single LCIA router are only 61,186 μm^2^ and 3.668 mW, where the scheduler only occupies 1.51% of the router area. This makes it applicable for larger scale hardware SNN systems. The main contributions of this paper include:

(a) LCIA: A novel all-to-all interconnection architecture is developed to connect paired input and output nodes of multi-layer SNNs, and a compact scheduler is designed to arbitrate the input channels.
(b) Experimental results and detailed performance analysis demonstrate the efficient routing capability of LCIA under different spike patterns.
(c) The low hardware area of the LCIA maintains the scalability of the hardware SNN systems.

The rest of the paper is organized as follows: ˆsection 2 provides a summary of related work. Section 3 discusses the proposed LCIA in detail. Section 4 gives experimental results and performance analysis of the LCIA under different spike patterns. Section 5 discusses the hardware implementation of LCIA using a field-programmable gate array (FPGA) technology and provides an area and power consumption comparison with previous works. Section 6 concludes the paper and provides the plans for future work.

## Related Works

In this section, a brief review of various SNN implementations is presented. Particularly, current NoC-based interconnected strategies for hardware SNN implementations are discussed, and their suitability in supporting SNN hardware implementations are also highlighted.

### Summary of Various SNN Implementation Approaches

Various approaches have been explored for SNN implementation, including software, application-specific integrated circuit (ASIC), GPU, field-programmable gate array (FPGA), and so on. Current software approaches based on the traditional von Neumann computer paradigms are too slow for the SNN simulations and suffer from the limited scalability as the SNN systems are inherently parallel (Furber et al., 2013; Lagorce et al., 2015). Another approach is GPU-based architecture, which provides a fine-grained parallel architecture and archives a computing acceleration compared to the CPU-based solution, e.g., the approaches of Fidjeland and Shanahan (2010) and Wang et al. (2011) proposed the simulation frameworks for the SNNs on the GPU platform. However, the main drawback of this technology is that the high-end computers (GPUs included) are generally costly in terms of power consumption (Moctezuma et al., 2015; Kwon et al., 2018). In addition, it has limited memory bandwidth, which constraints the data transfer rate between the GPU and CPU (Nageswaran et al., 2009). They are currently the major drawbacks for realizing large-scale SNN systems. Recently, researchers have attempted to use custom hardware to design the SNNs, e.g., ASIC and FPGA devices. For the former, many approaches have been proposed, e.g., TrueNorth chips (Merolla et al., 2014; Akopyan et al., 2015), a neuromorphic analog chip (Basu et al., 2010) and Neurogrid, a large-scale neural simulator based on a mixed analog-digital multichip system (Benjamin et al., 2014). The main disadvantage of using ASIC devices is the high cost for the development and chip manufacturing as a tiny change would lead to a new development cycle (Pande et al., 2013). For the latter, the ability to reconfigure FPGA logic blocks has attracted researchers to explore the mapping of SNNs to FPGA (Graas et al., 2004; Upegui et al., 2005; Morgan et al., 2009; Cawley et al., 2011; Ang et al., 2012; Pande et al., 2013). For example, the ENABLE machine, a systolic second level trigger processor for track finding, was implemented based on a Xilinx FPGA device in the approach of (Klefenz et al., 1992). It used regular interconnection for the communications between building blocks. A reconfigurable point-to-point interconnect is proposed in the approach of Abdali et al. (2017) to provide a lightweight reconfigurable interconnect system. However, the previous work in Harkin et al. (2009) and Carrillo et al. (2012) have highlighted the challenges of supporting the irregular communication patterns of SNNs due to its Manhattan style interconnections. In addition, it has been demonstrated that the topology of the bus is not scalable for the hardware SNNs as the number of required buses is proportional to the number of neurons (Carrillo et al., 2012). Therefore, it is necessary to look into new full-custom hardware architectures to address the interconnection problems of hardware SNNs.

### Current NoC-Based Spiking Neural Network Approaches

In the hardware SNNs, the interconnection strategy of NoC is used to support the communication requirement of SNNs. The advantages of using NoCs for SNNs have been discussed in previous works (Schemmel et al., 2008; Harkin et al., 2009; Carrillo et al., 2012, 2013; Painkras et al., 2013; Liu et al., 2015). The following text summarizes current state-of-the-art NoC-based hardware SNN architectures.

The SpiNNaker platform was proposed in Jin et al. (2010), which is based on a multiprocessor architecture. It uses ARM968 processor cores as the computational elements and a triangular torus topology to connect the processors. It has been used for the simulations of a cortical microcircuit with ∼80,000 neurons and 0.3 billion synapses (Van Albada et al., 2018). The FACETS in Schemmel et al. (2008) was based on a 2D torus which provided the connection of several FACETS wafers. Some routing architectures based on two-dimensional (2D) mesh were proposed in the approaches of Harkin et al. (2009); Carrillo et al. (2012), and Liu et al. (2015). Additionally, a hierarchical NoC architecture for hardware SNN was proposed in the approach of Carrillo et al. (2013), which combined the mesh and star topologies for different layers of the SNNs. Most of these systems used either the baseline or some variations of the well-known mesh topology to connect the neurons together. However, for a large scale SNN, when the size of NoC increases, the average communication latency increases due to the large number of indirect connections of the mesh topology (Mohammadi et al., 2015). For instance, when a spike event needs to be forwarded to the neurons in the next layer of SNN, some intermediate nodes are required for the transmissions, which increases delay. In addition, the multiple layer SNNs are generally based on fully connected communications. To map it to the regular topology leads to a high hardware area overhead of the interconnection fabric which constraints the scalability. Therefore in this paper, the LCIA is proposed to provide an efficient communication mechanism for the SNNs with a low hardware cost and a high scalability.

## LCIA

In this section, an ENA tile architecture for neuron node in our previous work (Wan et al., 2016) is used as an example for the hardware SNNs. The proposed low cost interconnection architecture (LCIA) strategies are presented in detail. The all-to-all interconnected architecture and the efficient scheduling mechanism are also outlined.

### The ENA Tile Architecture

**FIGURE 1:**
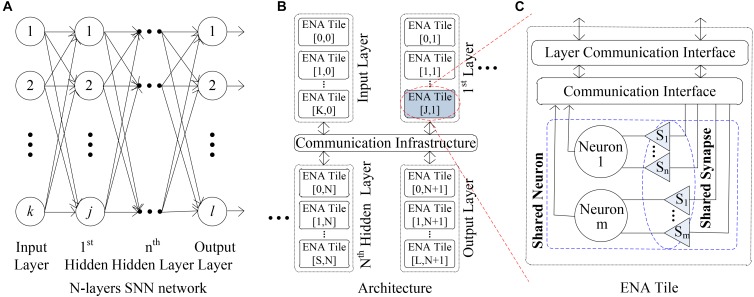
ENA overview. **(A)** N-layers SNN network. **(B)** Architecture. **(C)** ENA tile.

In general, the SNN is a multiple-layer network that includes an input/output layer and one or several hidden layers (Wang et al., 2015). Figure 1A illustrates a typical SNN where input, first hidden and output layers include *k*, *j*, and *l* neurons, respectively. Each neuron in the pre-layer is connected to all neurons in the next layer by the synapses. In our previous work (Wan et al., 2016), the ENA was designed for the hardware implementation of neuron node. It can accommodate a group of neurons in one layer of SNN. Figure 1B shows that the ENAs can be connected by a global communication infrastructure to realize a large scale SNN system. In particular, each ENA has the capability to accommodate up to ∼18,181 neurons and synapses in one facility. If the number of neurons in one layer is more than that, multiple ENAs can be used together, e.g., the input layer includes a total *K+1* ENAs (i.e., ENA[0,0] to ENA[K,0]) in Figure 1B. The ENA utilizes a computing resource sharing mechanism at two levels (i.e., synapse and neuron) to reduce the required computational resources, as shown by Figure 1C. The aim of this paper is to propose the low cost interconnection architecture for the multi-layer SNNs, where the ENAs (Wan et al., 2016) are used as an example for neuron nodes. Inside a single ENA the neurons can communicate with each other locally. Note that the LCIA is not constrained to the ENAs and can be applied to any other layer-based SNN hardware systems especially where the processing element in the NoC includes a large number of neurons. Only several LCIAs are required for the interconnections of the normal scale SNNs. For the very large SNNs, a hierarchical structure can be considered where the entire LCIA can connect to a node of a high level LCIA. Therefore, the proposed LCIA can maintain the network scalability. The details are discussed in the following sections.

### Spike Patterns and Traffic Loads

**FIGURE 2:**
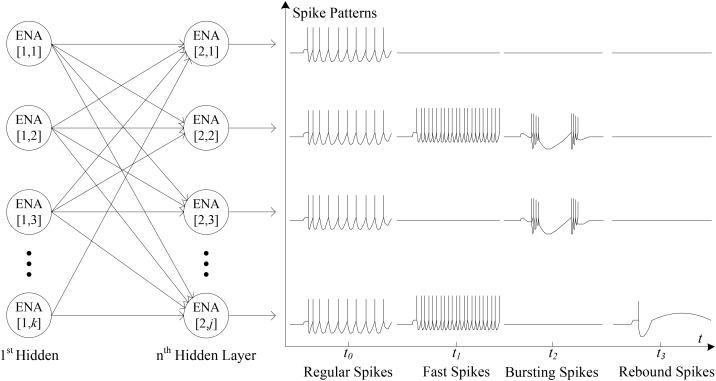
The typical spike patterns.

The approach of Carrillo et al. (2012) introduced the concept that the spike forms of spiking neurons are highly irregular and have a major impact on the latency of packet delivery and ultimately may lead to traffic congestion. Figure 2 shows an example of the typical spike forms, including the regular spikes, the fast spikes, the bursting spikes and the rebound spikes (Carrillo et al., 2012). Note the fundamental characteristics of these spike forms: (a) *the regular spikes*: every neuron from the same layer generates spike events, regularly; (b) *the fast spikes:* the high-frequency spike events may suddenly be generated by some of all neurons; (c) *the bursting spikes:* one or more neurons occasionally output some bursting spike clusters; (d) *the rebound spikes:* one or several spikes are randomly generated by a few neurons.

Various spike scenarios can be presented within an SNN application. In the meantime, when the SNN scales, the network connectivity becomes sparse (Jordan et al., 2018) which leads to unbalanced traffic load across the NoC. To maintain good performance under different spike scenarios and traffic balances, the routing architecture of the NoC should be efficient for the various scenarios. This architecture is introduced with more details in the next sections.

### LCIA Overview

**FIGURE 3:**
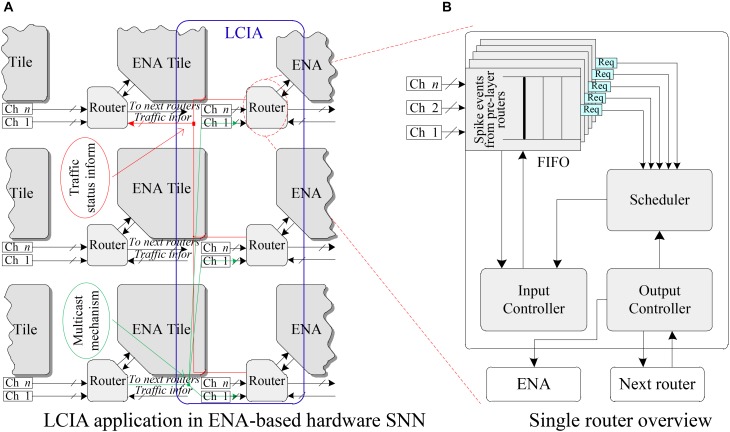
LCIA and its connection. **(A)** LCIA application in ENA-based hardware SNN. **(B)** Single router overview.

In this work, the LCIA is proposed to efficiently forward the spike events for the SNNs. The interconnections between the LCIAs and ENAs are given in Figure 3A. The LCIA is an all-to-all connection method that takes the paired input and output nodes of multi-layer SNNs as the source and destination of connection. A novel NoC router is used as the fundamental unit of LCIA. Each ENA connects to the local port of a router, and the router has a one-to-all connections (broadcast, e.g., the green lines in Figure 3A) to the ones in the next layer. The traffic information (red lines) are used for transmitting the traffic status information such as busy, congested, so on. In this example, each router has *n* input channels (Chs) that are shown as parallel connections for receiving the outputs of the routers in previous layer. The LCIA can also be used for the multicast communications between the neurons which can be achieved by adding a mask section in the packet layout. If the destination routers have the matched mask, they receive and forward the packets otherwise the packets are discarded. After the ENA generates spike packets, the router forward these packets to the routers in next layer. The routers in the next layer may receive multiple spike packets from different ENAs, requiring the router to have the ability to arbitrate the various input traffic.

The overview of a single router architecture is shown in Figure 3B, where a FIFO, an Input Controller, a Scheduler module and an Output Controller are included. When multiple spike packets arrive in the input channels, the FIFOs are in charge of caching these spike packets temporarily. The scheduler is used for scheduling these spike events. The input and output controllers are responsible to control the packet read and output operations, respectively. As the router and ENA have the same architecture in the SNN, only the structure and functionality of a single router are presented in detail in the following sections.

### Efficient Scheduling Policy

In the routers of the hardware SNN, an effective scheduler should find requests rapidly and make the arbitration decision in a short time period. In the meantime, it should also provide fair and equal services to the input traffic requests (Carrillo et al., 2010). Several scheduling policies have been proposed (Dally and Towles, 2004), i.e., the first-come first-serve scheduler and the round-robin scheduler. A first-come first-serve scheduler gives the highest priority to the first event that occurs. Thus, it is efficient for the spike scenario where only one or a few ports are busy (e.g., the rebound spike pattern), as the router does not spend extra clock cycles servicing inactive or unused ports. However, it’s not feasible for the bursting spikes since the packet waiting time from inactive ports increases as the arbiter priority is given only to the first requested port (Carrillo et al., 2012). However, a scheduler based on the round-robin arbitration policy exhibits a strong fairness, since it allocates equal priorities to all ports (Zheng and Yang, 2007). It is good for the regular spike scenarios where all router ports have data transmission requests. However, since it cannot skip the inactive ports, the router latency is proportional to the number of inactive ports of the router (e.g., some inactive ports can be found in the fast, bursting and rebound spike patterns). Therefore, an efficient scheduling policy is proposed in this work which combines advantages of the aforementioned two arbitration approaches. The scheduler services only those ports that require information transmission, avoids wasting clock cycles on inactive or unused ports, and services all active ports successively based on the fairness mechanism without starvation.

**FIGURE 4:**
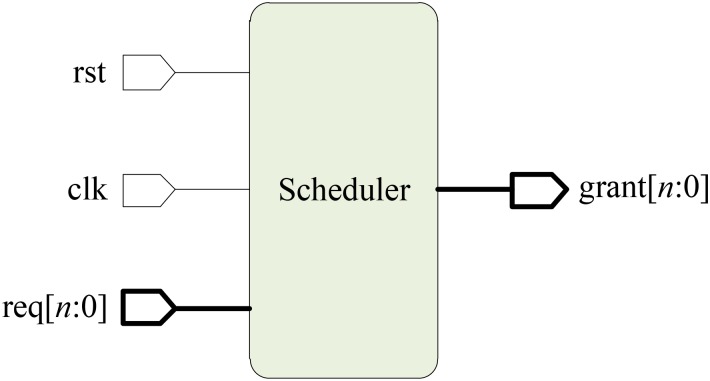
Scheduler block diagram.

The scheduler block diagram, illustrated in Figure 4 to handle *n* different requests, includes reset, clock, and a *N*-bit require and grant signal ports. The require information is given by each of the “data present” signals provided by the FIFOs. The scheduler can know when and where a spike event has occurred by using these signals, then the grant signal is generated.

**FIGURE 5:**
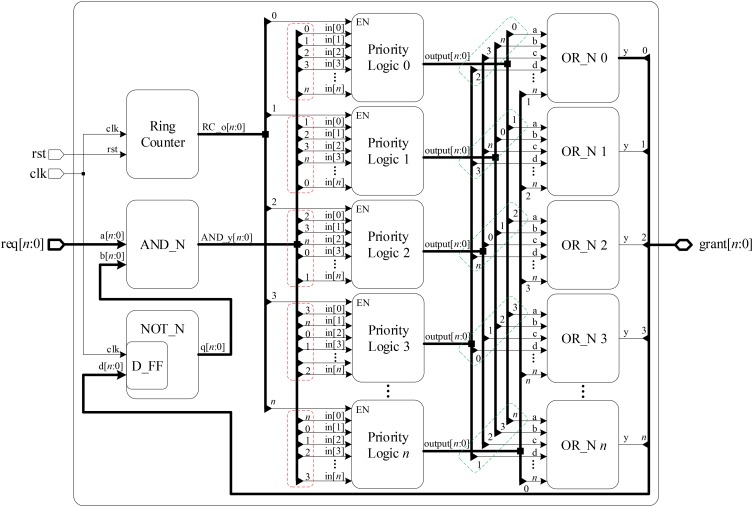
Logic diagram of the *n* × *n* scheduler block.

Figure 5 shows the logic diagram for an *n* × *n* scheduler block. The scheduler consists of an *n*-bit ring counter, *n n*-input OR gates, *n* priority logic blocks, an AND gate and an NOT gate, where *n* can be set according to the requirement, e.g., *n* = 4 for four require signals. Note that *n* parallel _n × n_ priority logic blocks are included, and each priority logic block is implemented using combinational logic whose truth table is illustrated in Table 1. The input priorities are set in descending order from input *0* to *n* in Priority Logic 0 through *n*, i.e., inputs “in[0]” and “in[n]” have the highest and lowest priorities, respectively. In addition, the connection sequences of request signals are varied in the different priority logic blocks, see the red rectangles in Figure 5. An *n*-bit Ring Counter is used to implement the polling operation between different priority logic blocks. The output of the Ring Counter is rotated after each clock cycle, e.g., for the 4-bit width, its output is from _N × N_ to _(1000)_2__ after one rotation where the default value is _(0001)_2__ after the reset. The output of the ring counter is used as the enable signal of the priority logic blocks. It allows one priority logic block to be enabled in turn, and this enabled priority logic block generates the grant signals. For the polling mechanism, each priority logic block must wait no longer than (*n*-1) time slots. The time is allocated to the other chosen priority logic block, until it receives the enable information in the next time slot. This protocol guarantees a dynamic priority assignment to requestors without starvation.

**Table 1:** Truth table of a N × N priority logic block.

Input						Output				
EN	in[0]	in[1]	in[2]	in[3]	in[*n*]	output[0]	output[1]	output[2]	output[3]	output[*n*]
0	×	×	×	×	×	0	0	0	0	0
1	1	×	×	×	×	1	0	0	0	0
1	0	1	×	×	×	0	1	0	0	0
1	0	0	1	×	×	0	0	1	0	0
1	0	0	0	1	×	0	0	0	1	0
1	0	0	0	0	1	0	0	0	0	1

**FIGURE 6:**
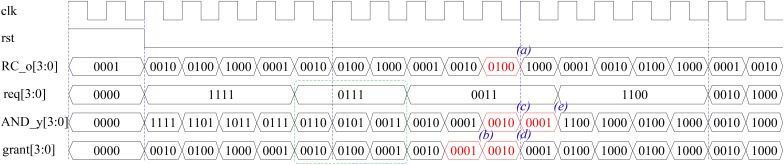
Simulation results of the scheduler.

To illustrate the details, suppose 4 ENA neuron nodes are connected to four routers as slave elements where four request signals are required for the interconnection. The simulation results of the scheduler are shown in Figure 6. In this example, the output port of the Ring Counter (RC_o), the request signal (req), the output port of the AND gate (AND_y), and the grant signal (grant) are included to illustrate the working mechanism of the scheduler. Assume that the output of the Ring Counter is _(0100)_2__ at one time point as shown by time (a) in Figure 6. It means only Priority Logic #2 is enabled, and only ENA #0 (req[0]) and ENA #1 (req[1]) request to transfer the spike events at this clock cycle (i.e., Req[3:0] is_(0011)_2__). In Priority Logic #2, the connection of “in[0]” (i.e., req[2] from ENA #2) has the highest priority, as “req[3]” is connected to “in[1]” of Priority Logic #2, ENA #3 has the second highest priority. Since ENA #2 and #3 do not make a request, the connection of “in[2]” (i.e., req[0] from ENA #2) has the next level priority. However, the req[0] has been granted at the previous cycle, see time point (b). Thus the corresponding request bit is shielded by an NOT gate and an AND gate in the next clock cycle of time point (c). Finally, only “req[1],” which is connected to “in[3]” of the Priority Logic #2, is granted, see time point (d). The ENA #1 is granted to output a spike event, i.e., the Input Controller reads a spike packet from its corresponding FIFO buffer, and then this grant information as a feedback signal is sent to a D flip-flop within the NOT gate. The feedback signal is delayed one cycle by the D flip-flop. The delayed feedback signal is sent to an NO gate, and its output is transferred to an AND gate. The request signal and feedback information are operated by the AND gate to shield the previous grant port. Note the output result of the AND gate (AND_y) is labeled by (e) in Figure 6 where the previous grant port has been shielded and the result is taken as a new input for the next circulation. The proposed scheduler services only the input ports that contain information, avoids wasting clock cycles for inactive or unused ports, where all active ports are serviced in turn based on the fairness mechanism without starvation. For example, the green section in Figure 6 shows that the require signal Req[3:0] is “0111,” the grant signal Grant[3:0] outputs the grant information of “0010,” “0100,” and “0001” in turn and skips the port without requirements. In addition, the first output is “0010” because the requirement of “0001” has been serviced in the previous clock cycle.

### LCIA Structure and Its Working Mechanism

**FIGURE 7:**
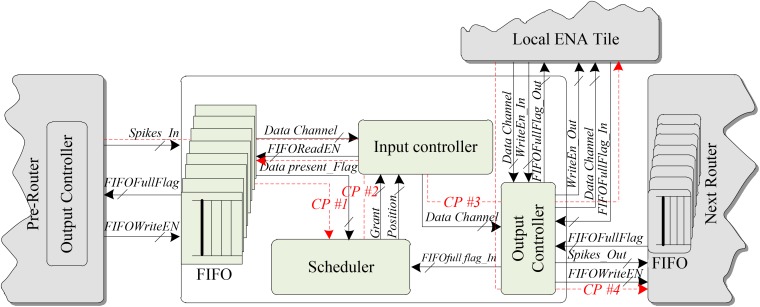
LCIA structure.

To understand the structure and the working mechanism of the LCIA, an example of a data transmission scenario based on a single router is presented in Figure 7. The proposed LCIA is an all-to-all interconnection architecture based on multiple routers, and a single router is used to introduce the working mechanism of LCIA, as other routers have similar working flows. Figure 7 shows the connections between the two routers in the LCIA and the local ENA tile. The following sub-blocks are inside the LCIA: (a) An FIFO component. It is used to store the spikes for different ENAs temporarily, and its depth grows linearly with the number of ENAs; (b) The Scheduler. It is used to make the arbitration decision for various spike events; (c) Input controller. After the Scheduler makes the grant result, the corresponding spike is granted for transmission from the Input Controller; and (d) Output Controller. It is used to control the packet forwarding processes.

When the packets from the pre-layer router arrive, the FIFOs in LCIA are allocated to store various packets temporarily if the output channel is busy and cannot forward packets immediately. Then, the Scheduler is used to make the arbitration decision according to the request information from the “data present” signal of the FIFO, which is shown by the communication path (CP) #1 in Figure 7. After completing the arbitration, the Scheduler generates the grant information to the Input Controller, as shown by CP #2. Then, the Input Controller converts it into a “read enable” signal of the FIFO, which is used to enable the read data port of the FIFO. The Input Controller reads the corresponding packets in relevant FIFOs and transfers to the Output Controller, which is then forwarded to the local ENA neuron node, as shown by CP #3. The spike events from the local ENA are forwarded to next-layer routers by the output controller, as shown by CP #4. In addition, the Output Controller prejudges its traffic status based on the “full” status signal of FIFO before transferring the packet information to the local ENA node or the next router. If the traffic is not congested (i.e., the “full” signal of FIFO is invalid), the packet continues to transfer, otherwise the transmission is waiting. Various traffic statuses probably cause packet latency jitters. This can be addressed by adding time stamp to the packet, where the neuron nodes calculate the membrane potential after all the synaptic information are received (i.e., the activities are synchronized). In addition, research shows that other form of spike-timing-dependent plasticity (such as endocannabinoid-plasticity) is highly resistant to jitter (Cui et al., 2018), which can be considered as an alternative learning rule.

## Methodology and Experimental Results

This section outlines the methodology used in performing experiments and presents results for the performance of the LCIA under different spike scenarios.

### Methodology of Evaluation

**FIGURE 8:**
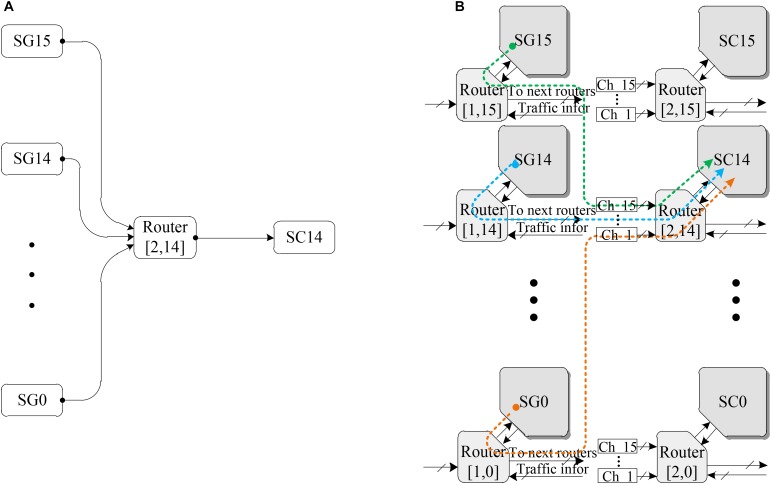
The neural network structure using the LCIA. **(A)** The interconnections of the router (Gerstner and Kistler, 2002; Schuman et al., 2017). **(B)** A 16 × 2 array of NoC routers.

The spike patterns of SNNs are highly irregular, according to the description in section 3.2. These irregular scenarios can have a major impact on the latency of packet delivery and additionally may lead to traffic congestion (Carrillo et al., 2010). Thus, the key aspect of the performance verification of the proposed LCIA routing architecture is to analyze how LCIA can guarantee effective routing capabilities (i.e., throughput) under various spike patterns. Considering the spike packet layout illustrated in Figure 2, the spike event generator (SG) and the spike event counter (SC) as the spike packet source and throughput calculation module from the approach of Carrillo et al. (2012) are employed in this paper to evaluate the performance of the LCIA. A VHDL co-simulation framework is presented in Figure 8. It has 16 × 2 array of LCIA-based routers as shown by Figure 8B where each router is connected to all the nodes in the previous layer and the local SG, e.g., Figure 8A shows that 16 SGs are attached to the input ports of an router R (Gerstner and Kistler, 2002; Schuman et al., 2017) and one output port is connected to the SC14. The SGs and SCs are attached to the input and output NoC router ports, respectively.

According to the description in section 3.2, spike patterns include regular, fast, bursting, and rebound spikes. In this experiment, various spike patterns can be simulated by the architecture in Figure 8, and this method is widely used to evaluate the performance of the hardware SNN router (Carrillo et al., 2012, 2013; Liu et al., 2015). The different spike injection rates (SIRs) can be simulated by changing the time interval between the spikes. The SIR refers to the rate at which spike packets are injected into the router. For any given single node router in the SNN, the number of injected spike packets per clock cycle is equal to SIR and has the range of_0 < SIR ≤ 1_. For example, if SIR = 0.2, the node sends 0.2 packets per clock cycle, i.e., 2 packets every 10 clock cycles. The different spike patterns can be simulated by controlling the SGs which are the inputs of the router (Gerstner and Kistler, 2002; Schuman et al., 2017) as well as the SIRs. For instance, the bursting spike pattern in Figure 2 can be simulated by the following setting: only two of the 16 SGs (SG0 to SG15) are enabled to generate spike packets, and each enabled SG uses a high spike injection rate (e.g., SIR = 0.5). In addition, if all 16 spike event generators SG0 to SG15 are enabled, a typical regular spike pattern can be simulated.

### Experimental Results

**FIGURE 9:**
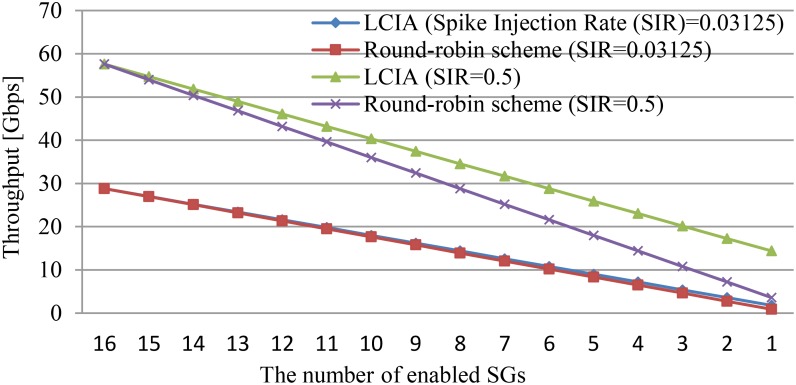
Relationship between the number of enabled SGs and the throughput at different SIRs.

This section presents the results from experiments on assessing how the LCIA guarantees throughput under different spike patterns. The results between the number of enabled SGs and the throughput at different SIRs is shown in Figure 9. In this example, the total number of SGs is 16. The round-robin scheme is used as the benchmark. The results include spike scenarios of (1) the regular or rebound spike pattern, and (2) the fast or bursting spike pattern.

#### The Regular or Rebound Spike Pattern Scenario

In this scenario, the traffic load is light or medium where SIR = 0.3125 is employed (i.e., the SG generates 1 packet every 32 clock cycles) and all 16 SGs are enabled for modeling. The results in Figure 9 illustrate that the LCIA and the round-robin-based routers achieve almost same performance when all 16 SGs are enabled at SIR = 0.3125. However, for the rebound spike pattern, only a few routers out of 16 are active (e.g., the number of enabled SGs is 1 or 2). It can be seen that under this pattern, the proposed LCIA router can skip idle ports, which avoids wasting clock cycles and achieves a higher throughput than the round-robin-based router. This advantage becomes more significant in the following fast or bursting spike pattern scenarios.

#### The Fast or Bursting Spike Pattern Scenario

For this scenario, not all router ports generate spike events. That is, the number of SGs that generate the spike packets is only a small percentage of the total SGs, but the SIR is higher than the regular spike pattern. In this example, the SIR is set to 0.5 to produce a bursting or fast spike. Figure 9 shows that when the number of enabled SGs decreases, the throughput difference between LCIA and the round-robin scheme becomes larger, and the LCIA has a much higher throughput. For instance, if only two input channels receive the spike packets (two SGs enabled), which is a typical bursting traffic pattern, the LCIA has a 140% throughput improvement than the round-robin scheme. Therefore, the LCIA architecture can maintain system throughput under different spike patterns. It has the advantage of high throughput especially for fast or bursting spike patterns, as it can efficiently arbitrate the data requests without wasting time on the channels with no data present. Thus, the experimental results of Figure 9 show that LCIA is able to balance the traffic load of the hardware interconnected SNNs.

## Hardware Implementation

This section presents the methodology for implementing the LCIA in hardware and the results of area overhead and power consumption. The performance comparison with state-of-the-art approaches is also given. The hardware implementation is based on a Xilinx XC7Z020-CLG484 device. The router is implemented based on a 100 MHz system frequency and the 36-bit packet data width. The pre-layout area overhead and power consumption have been evaluated based on a Synopsys Armenia Educational Department (SAED) 90 nm CMOS technology.

### Hardware Implementation

**FIGURE 10:**
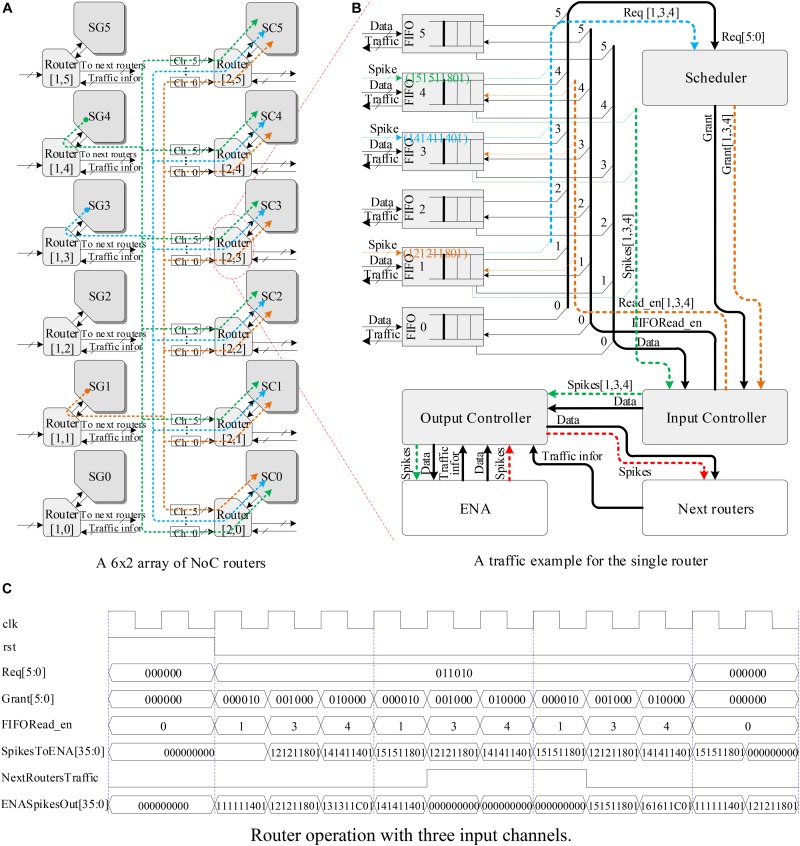
The hardware SNN system and router operations. **(A)** A 6 × 2 array of NoC routers, **(B)** a traffic example for the single router, and **(C)** router operation with three input channels.

Figure 10A shows a 6 × 2 array of NoC routers implementing the routing scheme of hardware SNN using the LCIAs. Only 3 out of 6 SGs (i.e., SG1, SG3, and SG4) are enabled to generate spike events. The spike packets from each SG are transmitted to all the routers in the next layer by a broadcasting method, and they are forwarded to all the SCs in the next layer. Figure 10B illustrates a traffic example for a single router R (Gerstner and Kistler, 2002; Carrillo et al., 2013) where 3 out of 6 input ports receive the spike events (X“121211801,” X“141411401,” and X“151511801”). Suppose that a worst case happens, i.e., the spike events from these three inputs arrive at the router ports at the same time. These spike events are forwarded in turn by the router R (Gerstner and Kistler, 2002; Carrillo et al., 2013) and follow four steps: (1) The spike events are saved temporarily by the FIFOs; (2) the Scheduler checks the request information from FIFOs (i.e., Req[1,3,4] in Figure 10B) and makes the corresponding grant decision; (3) the Input Controller reads the corresponding spike packets according to the grant decision; and (4) finally, these packets are forwarded to the local SCs by the Output Controller. In addition, if a spike event is from the local SG, it will be forward by the Output Controller to the routers in next layer.

Moreover, the runtime operation of this router is shown in Figure 10C. The grant decision is made in turn by *Grant[5:0]* and *FIFORead_EN* ports of the Scheduler with the request signal *Req[5:0]* of FIFO being “011010.” Only one clock cycle is consumed from receiving the FIFO request information to making the grant decision by the Scheduler. Next, the spike packets from the corresponding FIFOs are transmitted to the local SCs by the *SpikesToENA [35:0]* port of the Output Controller in turn. In addition, when the traffic statuses of the routers in the next layer are not congested, i.e., the *NextRoutersTraffic* signal is “0” as shown in Figure 10C, the spike events from the local SG will be forward by the Output Controller to the routers in the next layer.

### Performance Analysis

**FIGURE 11:**
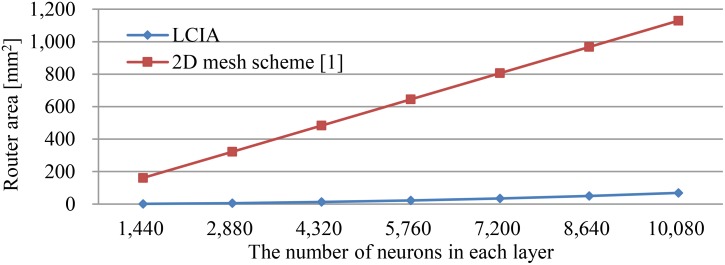
The comparison of router area overhead.

The scalability of the LCIA is analyzed as follows: (1) for the large-scale SNNs, the required routers, and ENAs increase with the number of neurons in each layer. The proposed LCIA supports a regular layout of the ENA tiles and neuron communication where the number of each router input port needs to be extended, e.g., n ENAs in each layer require an n input router. However, since each ENA can implement ∼180 neurons (Wan et al., 2016), e.g., for one layer with 1,440 neurons only 8 routers with 8 input ports are required, the increased port number of routers using LCIA does not limit the network size that can be implemented; and (2) Figure 11 shows the required router areas of two approaches, LCIA in this work and the approach of (Cios and Shields, 1997). In this work, the hardware area of a single router is less than the router in the approach of Cios and Shields (1997). In addition, the multiple neurons are included in each ENA rather than one router per neuron in the approach of Cios and Shields (1997). Thus, the LCIA can achieve much less area overhead for the large network compared to the approach of Cios and Shields (1997). Therefore, the proposed LCIA can maintain the scalability for the large neural network.

**FIGURE 12:**
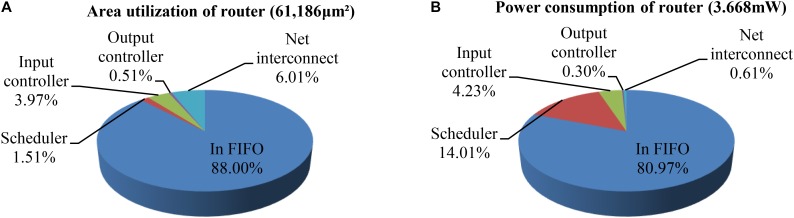
The area utilization and power consumption distributions per router. **(A)** Area utilization of router. **(B)** Power consumption of router.

For the scheduler in Figure 5, the critical path is from the request input to the generation of the grant, which determines that the maximum frequency is 280 MHz. Figure 12 shows the area utilization and power consumption of a single router including all modules – net interconnect, input FIFO, scheduler, and input/output controller. The results show that the total area overhead and power is 61,186 μm^2^ and 3.668 mW, respectively, where the FIFOs occupy the largest area and power (88 and 80.97%). These results are obtained using the Synopsys Design Compiler tool based on a SAED 90 nm CMOS technology, where a clock frequency of 100 MHz has been used. In general, buffered routers can reduce network contention (i.e., latency), but they are costly in terms of area overhead and power consumption (Agarwal et al., 2009). For the LCIA, if the buffer capacity is increased by one packet, the router area is increased by ∼17.6%. Thus, considering efficient router designs, it is important to find a trade-off between the capacity of input buffers and the performance. In previous work, some quantitative analysis has been conducted regarding the impact of varying the buffer capacity on the throughput, power consumption, and area utilization (Carrillo et al., 2012, 2013; Liu et al., 2015, 2016). For example, our previous work (Carrillo et al., 2012) shows that both of the power consumption and throughput increase proportionally to the buffer capacity; however, the change rate of former is more than double of the latter and the break-point is when buffer capacity is greater or equal to nine packets. Thus the buffer capacity should be less than nine. In the meantime, the power consumption and area utilization were also analyzed under various buffer capacities. Results showed that the power consumption and area utilization have the same change rates when the buffer capacity increases. Therefore considering the relationships between power consumption, throughput, and area utilization, a five-packet buffer capacity offers a good trade-off, and it is used in this work. For the large-scale networks, if the area utilization of buffers is too high, application-specific buffer space allocation technique (Hu and Marculescu, 2004) or bufferless router architecture (Moscibroda and Mutlu, 2009) could be used.

A comparison regarding the hardware overhead and power consumption of the proposed router with other existing approaches is shown in Table 2. The approaches in Zhang et al. (2008), Harkin et al. (2009), and Wang et al. (2010) have a relatively low area overhead, but they do not have the congestion-aware capability. The router areas for them are 68,000, 185,392, and 201,000 μm^2^, respectively. Other NoC routers (Carrillo et al., 2013; Liu et al., 2015, 2016) and the proposed LCIA are all equipped with a traffic congestion avoidance mechanism. SpiNNaker uses a communications NoC and a system NoC for the communication mechanism, where the former provides the communications for on and off-chip interprocessors and the latter handles the on-chip processor to memory/peripheral communications (Furber et al., 2013; Painkras, 2013). As its communications NoC handles large number of router entries, the hardware area is 9.7 mm^2^ based on an UMC 130 nm technology (Painkras, 2013). FACETS (BrainScaleS) (Schemmel et al., 2008; Schmitt et al., 2017) contains large number of analog neuron and synapse circuits, and it uses hierarchical buses and NoC routers for the inter/intra-wafer communications. The proposed LCIA provides a general communication infrastructure for the all-to-all interconnection in the neural network with a relative low hardware area, and it provides communications for customized neural network hardware systems. The approaches of Carrillo et al. (2013) are based on a hierarchical star topology. The approaches of Liu et al. (2015) and Liu et al. (2016) are based on a 2D mesh topology. Each router contains five input FIFOs for the North/E/S/W and local ports. For the fairness of comparison, five input ports are set in proposed LCIA. Based on the Xilinx XC7Z020-CLG484 device, the router uses 3,334 slide LUTs, 11,653 slice registers, 1,440 F7 Muxes, and 648 F8 Muxes. Based on the pre-layout results of SAED 90 nm technology, the area overhead and power consumption of the LCIA are 61,186 μm^2^ and 3.668 mW, respectively. The routers in the approaches of Carrillo et al. (2013) and Liu et al. (2016) have a higher power consumption. In addition, compared with the approach of Liu et al. (2015), the LCIA has a slightly higher power consumption, however, the hardware area overhead is much less than (Liu et al., 2015). Thus, comparing with other approaches, LCIA achieves a relatively low resource consumption.

**Table 2:** Router hardware overhead and power consumption comparison.

The approach	Congestion aware	Throughput (Gpbs)	Power (mW)	Area (μm^2^)	Device technology
Zhang et al., 2008	×	N/A	N/A	68,000	SXLIB 90 nm
Wang et al., 2010	×	N/A	N/A	185,392	SMIC 0.18 μm
EMBRACE Harkin et al., 2009	×	16	1.72	201,000	90 nm CMOS
H-NoC Carrillo et al., 2013	√	3.33	13.16	587,000	TSMC 65 nm
CG Liu et al., 2016	√	NA	16.172	237,115	SAED 90 nm
FG Liu et al., 2016	√	NA	27.266	267,756	SAED 90 nm
EDAR Liu et al., 2015	√	18	2.291	241,000	SAED 90 nm
This work	√	18	3.668	61,186	SAED 90 nm

## Conclusion and Future Work

A novel LCIA is proposed in this paper to provide a communication mechanism for the hardware SNN systems. The aim is to maintain efficient routing with a low hardware cost. This approach employs a NoC router as the fundamental unit for the SNN interconnections where an efficient scheduling policy is used to improve the communication efficiency between the neurons. Results show that the proposed LCIA is effective under various spike patterns, and the hardware overhead is relatively low enabling system scalability to be maintained. The future work will explore to further optimize the NoC routers.

## Author Contributions

YL, LW, and JL interconnected the architecture was developed, implemented, and evaluated. YL and LW wrote and revised the manuscript. JH and LM analyzed the performance of the proposed architecture and reviewed the manuscript. YC and XD investigated the arbitration mechanism of the router and reviewed the manuscript.

## Conflict of Interest Statement

The authors declare that the research was conducted in the absence of any commercial or financial relationships that could be construed as a potential conflict of interest.
